# Open versus closed intramedullary nailing of femur shaft fractures in adults: a systematic review and meta-analysis

**DOI:** 10.1007/s00264-023-05740-x

**Published:** 2023-03-03

**Authors:** Loay A. Salman, Abdallah Al-Ani, Mohammed F. A. Radi, Abedallah F. Abudalou, Omar M. Baroudi, Abdulla A. Ajaj, Mohamed Alkhayarin, Ghalib Ahmed

**Affiliations:** 1 Orthopedics Department, Hamad General Hospital, Hamad Medical Corporation, PO Box 3050, Doha, Qatar; 2 Nuffield Department of Orthopaedics, Rheumatology and Musculoskeletal Sciences, Botnar Research Centre, University of Oxford, Windmill Road, Oxford, OX3 7LD UK; 3 Office of Scientific Affairs and Research, King Hussein Cancer Center, Amman, Jordan; 4 Present Address: Department of Orthopaedic Surgery, Surgical Specialty Center, Hamad Medical Corporation, Doha, Qatar

**Keywords:** Open reduction, Closed reduction, Intramedullary nail, Femur, Fractures

## Abstract

**Purpose:**

This systematic review and meta-analysis aimed to compare the outcomes of open- versus closed-reduction and intramedullary nailing (IMN) of adult femur shaft fractures.

**Methods:**

Four databases were searched from inception until July 2022 for original studies that compared the outcomes of IMN following open-reduction versus closed-reduction technique. The primary outcome was the union rate; the secondary outcomes were time to union, nonunion, malalignment, revision, and infection. This review was conducted in line with PRISMA guidelines.

**Results:**

A total of 12 studies with 1299 (1346 IMN cases) patients were included, with a mean age of 32.3 ± 3.25. The average follow-up was 2.3 ± 1.45 years. There was a statistically significant difference in union rate (OR, 0.66; 95% CI, 0.45–0.97; *p*-value, 0.0352), nonunion (OR, 2.06; 95% CI, 1.23–3.44; *p*-value, 0.0056), and infection rate (OR, 1.94; 95% CI, 1.16–3.25; *p*-value, 0.0114) between the open-reduction and closed-reduction groups in favour of the latter. However, malalignment was significantly higher in the closed-reduction group (OR, 0.32; 95% CI, 0.16–0.64; *p*-value, 0.0012), whereas time to union and revision rates were similar (*p* = NS).

**Conclusion:**

This study showed that closed-reduction and IMN had more favourable union rate, nonunion, and infection rates than the open-reduction group, yet malalignment was significantly less in the open-reduction group. Moreover, time to union and revision rates were comparable. However, these results must be interpreted in context due to confounding effects and the lack of high-quality studies.

**Supplementary Information:**

The online version contains supplementary material available at 10.1007/s00264-023-05740-x.

## Introduction

Femur shaft fractures are among the most common fractures encountered in orthopaedic trauma practice, with an overall incidence of 10–21/100,00 persons per year [1, 2]. Of those, 40% occurred due to road traffic accidents and other high-energy trauma mechanisms [1, 2].

Due to the principal load-bearing role of the femur, femur shaft fractures are often associated with prolonged morbidity and extensive disability if improperly treated [3]. As a result, tremendous advances in treating femoral shaft fractures have been seen, with the gold standard for treatment remaining intramedullary nailing (IMN). While the standard routine insertion of IMN is done following closed fracture reduction, this is not always feasible due to fracture complexity, equipment’s availability, surgeon’s experience, and patient-related factors (obesity and polytrauma); thus, an open-reduction technique might be needed to achieve proper reduction alignment in some challenging cases [4].

In addition, several studies have shown that the open-reduction technique is associated with a higher risk of infection and lower union rates [5–7]. However, some of the setbacks were low power with small sample sizes and short-term follow-up. Therefore, high-quality evidence is needed to compare outcomes properly across both groups.

The purpose of this study was to compare the clinical and radiological outcomes of open- versus closed-reduction and IMN of such fractures. We hypothesize that there is no significant difference in outcomes and complication rates between patients treated with open-reduction and IMN versus those treated with closed-reduction and IMN.

## Materials and methods

This systematic review was conducted in accordance with the Preferred Reporting Items for Systematic Reviews and Meta-Analyses (PRISMA) guidelines [8]. A protocol registration was sought in advance on the International Prospective Register of Systematic Reviews (PROSPERO) with the registration number: CRD42023327089.

### Search strategy: outcomes of interest

PubMed/Medline, Web of Science, Google Scholar, and Cochrane library databases were searched from inception until July 2022 with the following keywords and their derivatives: Open AND closed AND reduction AND intramedullary nail AND femur shaft AND fractures. Two authors independently screened the search results based on the title and/or abstract. If any discrepancy arose, it was resolved by a discussion with a third senior author.

The union rate was the primary outcome and was defined as the radiological bridging callus formation across three out of four cortices with a painless fracture site. Time to union, non-union, malalignment, infection, and revision rates were all used as secondary outcomes of interest.

### Eligibility criteria

Inclusion criteria:

1. RCTs and observational studies comparing open versus closed reductions and intramedullary nailing of femoral shaft fractures
2. Studies with a minimum follow-up period of six months
3. Skeletally mature patients > 16 years

Exclusion criteria:

1. Studies reporting open and pathological fractures
2. Studies with proximal or distal femur (non-diaphyseal) fractures
3. Review articles, cross-sectional, case series, reports, and noncomparative studies
4. Studies missing essential data needed for analysis
5. Studies conducted on animals
6. Studies published in languages other than English

### Data extraction and items

Two independent authors used a pre-designed data collection sheet to extract data. The extracted demographic data included the first authors’ surnames, study year, design and country, the mean age of patients, number of participants, number of IMN cases (open vs closed), mean follow-up period, union rate, non-union, malalignment, time to union, operative time, infection and revision rate, statistical tests, and conclusion.

### Qualitative assessment (risk of bias)

Two authors assessed the methodological quality of the included studies using the Newcastle–Ottawa tool, which comprises three main elements: patient selection, comparability, and outcomes [9, 10]. A higher overall score indicates a lower risk of bias; a score of 5 or less (out of 9) corresponds to a high risk of bias. Rob-2 [11] tool was used to evaluate the included RCT.

### Quantitative analysis (meta-analysis)

A meta-analysis of eligible studies using R (version 4.0.2, R Core Team, Vienna, Austria, 2020) using the meta package (i.e., forest_meta, metacont, metabin, and metabias functions) was performed. Odds ratios (OR) and their associated 95% confidence intervals were expressed for dichotomous variables (e.g., rate of union). For continuous variables, standardized mean differences (SMD) and their associated standardized errors and deviation values were calculated for all eligible studies. In studies that have only provided median values (± range) or isolated mean values, their standard deviation was imputed per the guidelines of Cochrane (refer to Chapter 7.7.3.3) and the methods delineated by Shi et al. (2020), Walter et al. (2007), Luo et al. (2018), and Wan et al. (2014). Heterogeneity among effect sizes was evaluated using the *I*-squared statistic. Definitions for heterogeneity were adapted from the Cochrane handbook (> 25% mild, 25–50% moderate, > 50% severe). Both a funnel plot and Egger’s test of asymmetry were utilized to assess publication bias. Due to the low heterogeneity for the dichotomous variables, a common-effects model was utilized for the included studies; otherwise, a random-effects model was deployed.

## Results

### Search results

**Fig. 1:**
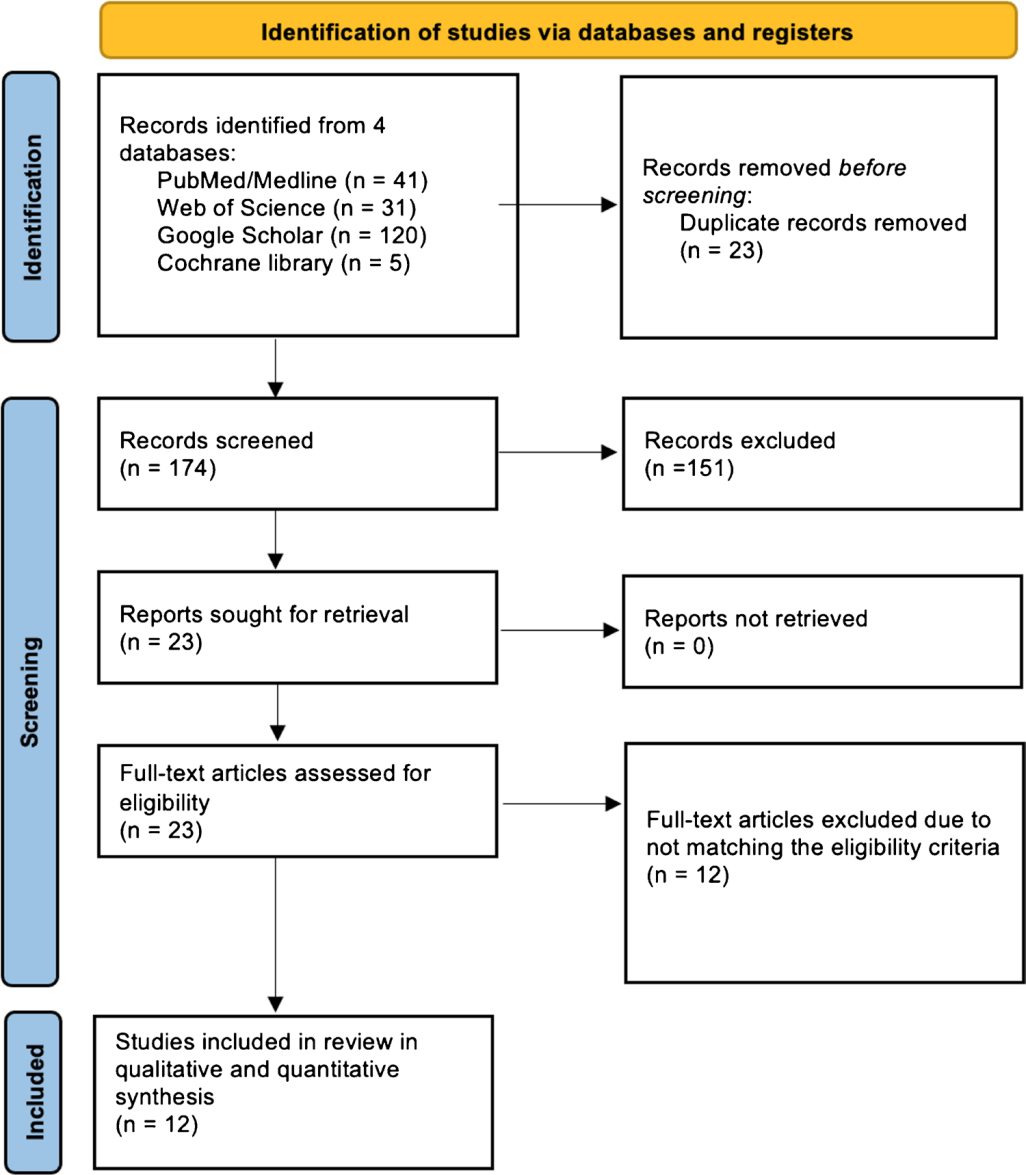
PRISMA flow diagram of record identification, screening, and selection in meta-analysis

Rayyan AI website was used to manage the literature search results [12]. Searching the databases yielded 197 articles, and after removing 23 duplicates, 174 records were screened by title and abstracts, of which 151 were excluded. A total of 23 papers were eligible for a full-text review. As a result, 12 studies met the eligibility criteria and were included in the qualitative and quantitative syntheses. The PRISMA flowchart is displayed in Fig. 1.

### Studies characteristics

Twelve studies (1331 femur shaft fractures) were included in this meta-analysis, with a mean patient age of 32.3 ± 3.25. The mean follow-up was 2.3 ± 1.45 years. One randomized clinical trial was included, six studies were prospective cohorts, and five were retrospective. Ten studies were used to analyse union rates, eight studies for time to union, malalignment, and revision, and nine studies reported on non-union and infection rates. The characteristics of the included studies are summarized in Table 1.

**Table 1:** A summary of baseline study characteristics

Author, year	Design, LoE	No. of participants	IM nails (open/closed)	Mean age (range)	Mean FU (years)	Primary outcome	Secondary outcomes	Statistical test	Conclusion
Leighton 1986 [13]	Retrospective, 3b	126	130(65/65)	28	4.16	Functional outcomes (ability to walk unaided)	Complications and failures	Chi^2^	No difference
Gharahdaghi 2007 [14]	Cohort, 2b	120	136(55/81)	36.2	2	Functional outcome (time to full WB)	Radiological union, complications, and failures	*T* test, Fisher test	In closed: shorter duration to union, lower non-union, higher malalignment
Tahririan and Andalib 2014 [15]	Cohort, 2b	48	47(23/24)	27.3(16–62)	NR	Union rate	Time to union, non-union, infection, revision	*T*-test	Mean time to union is significantly better in closed
Kimmatkar 2014 [4]	Cohort, 2b	272	272(110/162)	35.8(16–68)	3	Functional outcome (time to full WB, knee ROM)	Complications (nonunion, rotational deformity, malunion)	*T* test	In closed FWB earlier and higher malalignment (rotational deformity). Higher non-union in open group
Seetharamaiah 2015 [16]	Cohort, 2b	106	106 (49/57)	18–45	1	Functional outcome (Thoreson’s criteria)	Radiological union, complications (SSI, shortening, malalignment, delayed union)	Mean, Chi^2^	No difference
Chaudhary 2017 [17]	RCT, 2a	80	80(40/40)	Age categories given	4	Union rate	Blood loss, infection, functional outcome, operative time, complications	Mean, Incidence rate	Operating time, blood loss, and infection are higher in the open group. Cost of treatment and union is comparable in both groups
Kumar and Kumar 2018 [18]	Cohort, 2b	50	50 (25/25)	31(18–57)	NR	Functional outcome (Thoreson’s criteria)	Radiological union, complications (infection, shortening, malalignment, implant removal or breakage)	NR	No difference
Kisan and Samant 2018 [19]	Retrospective, 3b	64	74(28/46)	NR	4.33	Time to union	Operative time, complications (Infection, Nonunion, Malalignment deformity, pain, Implant failure, nerve injury, refracture)	Percentage, incidence rate	Higher avg time to union operative time and infection rate in open group. Higher rate of malalignment in closed
Ghouri 2020 [20]	Retrospective, 3b	110	110(37/73)	32.6	1	Union rate	Operative time, infection rate	*T*-test, Chi^2^, Fisher exact	No difference
Telgheder et al. 2020 [21]	Retrospective, 3b	107	107(37/70)	35.3(17–87)	1.2	Union rate	Time to union, complication, operative time	ANOVA, Chi^2^	No difference
Haq SN, 2020 [22]	Retrospective, 3b	116	116(54/62)	31(19–57)	1	Union rate	Nonunion, infection, time to union, operative time	Chi^2^	Closed had earlier union, better union, and infection rates
Nandhimandalam 2021 [23]	Cohort, 2b	100	118(56/ 62)	33.5(18–74 years)	1	Time to union	Non-union, malalignement, infection rate, revision, operative time	Paired* t*-test	Significant difference in mean surgical duration and c-arm shoots. No difference between time for union, rate of union, functional results and incidence of superficial or deep infection

*LoE*, level of evidence; *IM*, intramedullary; *FU*, follow-up; *NR*, not reported; *RCT*, randomized controlled trial

### Quality assessment (level of evidence (LoE) and risk of bias)

Based on the OCEBM criteria [24], one study was level 2a, six studies were level 2b, and five were level 3a (Table 1), with an overall grade B of recommendation assigned to the review [25]. The Newcastle–Ottawa scores of all 11 observational studies ranged from 4 to 7, with an average of 6 ± 1, indicating an acceptable overall risk of bias (Table 2). However, using Rob-2 assessment tool, the RCT by Chaudry et al. had a high overall risk (Table 3). While five (41%) of the included studies reported high bias risk, the remaining nine (59%) were of fair quality. A summary of the qualitative assessment, according to the Newcastle–Ottawa scale, is shown in Table 2.

**Table 2:** Risk of bias was assessed using the Newcastle–Ottawa Scale. A higher overall score indicates a lower risk of bias; a score of 5 or less (out of 9) corresponds to a high risk of bias

Study	Selection	Comparability	Outcome	Total score
Gharahdaghi 2007 [14]	***	0	**	5
Ghouri 2020 [20]	***	**	*	6
Kimmatkar 2014 [4]	**	*	*	4
Kisan and Samant 2018 [19]	***	*	*	5
Leighton 1986 [13]	***	**	**	7
Nandhimandalam 2021 [23]	***	**	**	7
Seetharamaiah 2015 [16]	**	**	*	5
Kumar and Kumar 2018 [18]	***	**	**	7
Tahririan and Andalib 2014 [15]	**	**	**	6
TeLgheder et al. 2020 [21]	***	**	**	7
Haq SN 2020 [22]	***	**	**	7

**Table 3:** Rob-2 risk of bias assessment tool used to evaluate included RCTs

Study	Randomization process	Deviations from intended interventions	Missing outcome data	Measurement of the outcome	Selection of the reported result	Overall Bias
Chaudhary P, 2017	H	S	S	H	S	H

*H*, high bias; *S*, some bias

### Union rate

**Fig. 2:**
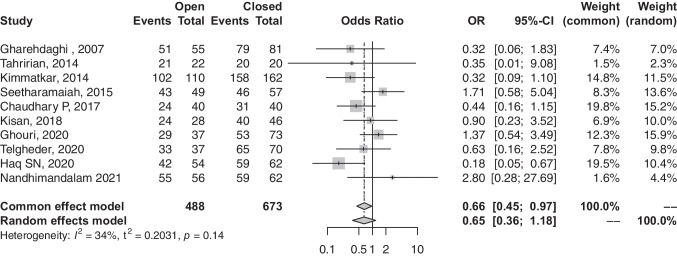
Forest plot comparison of the union rate between open and closed groups. OR, odds ratio; CI, confidence interval; Event, number of united fractures; total, all treated fractures

A total of ten articles investigated rate of union among patients undergoing open- and closed-type reductions. Our analysis demonstrated that patients undergoing open-reduction surgeries are associated with a lower probability of union than their closed counterparts (OR, 0.66; 95% CI, 0.45–0.97; *p*-value, 0.0352) (refer to Fig. 2).

### Time to union

**Fig. 3:**
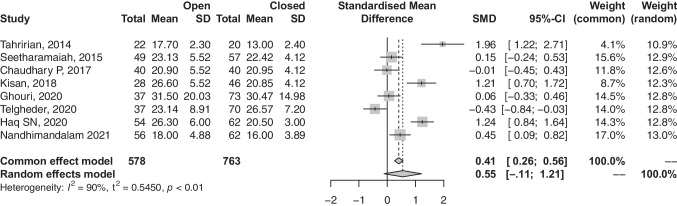
Forest plot comparison of the time to union between open and closed groups. CI, confidence interval; SD, standard deviation; CI, confidence interval; SMD, standardised mean difference; total, all treated fractures

Eight articles provided relevant data on the time taken for completion of union. Heterogeneity among articles was high (*I*^2^, 89.7%; *p*-value, < 0.001); thus, a random-effects model was utilized. Differences in time to union were insignificant among participants undergoing open- and closed-reductions (SMD, 0.41; 95% CI, − 0.11 to 1.21; *p*-value, 0.0906) (refer to Fig. 3).

### Non-union rate

**Fig. 4:**
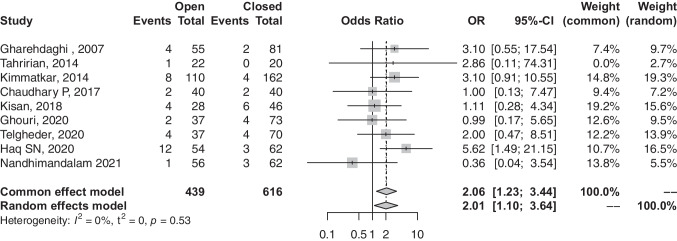
Forest plot comparison of non-union between open and closed groups. OR, odds ratio; CI, confidence interval; Event, number of non-unions; total, all treated fractures

In terms of direct non-union rates, a total of nine studies reporting on incidence of non-union across open- and closed-reduction surgeries. Participants undergoing open-reduction were associated with less favourable outcomes compared to closed-reduction in terms of non-union rates (OR, 2.06; 95% CI, 1.23–3.44; *p*-value, 0.0056) (refer to Fig. 4).

### Malalignment rate

**Fig. 5:**
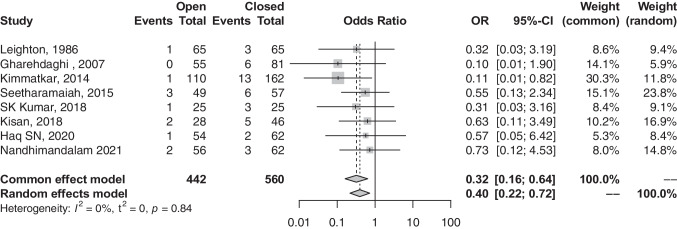
Forest plot comparison of malalignment between open and closed groups. OR, odds ratio; CI, confidence interval; Event, number of malalignments; total, all treated fractures

Participants undergoing open-reduction surgeries were associated with a lower probability of experiencing post-operative malalignments (OR, 0.32; 95% CI, 0.16–0.64; *p*-value, 0.0012) (refer to Fig. 5). Heterogeneity among the eight articles reporting on malalignment was low (*I*^2^, 0.0%; *p*-value, 0.8351).

### Infection rate

**Fig. 6:**
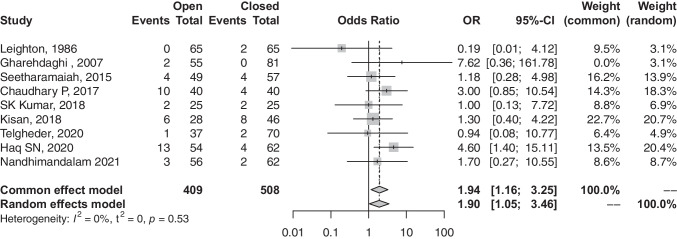
Forest plot comparison of infection rate between open and closed groups. OR, odds ratio; CI, confidence interval; Event, number of infections; total, all treated fractures

Probability of post-operative infections among patients undergoing open reduction was significantly higher than that of their closed-reduction counterparts (OR, 1.94; 95% CI, 1.16–3.25; *p*-value, 0.0114) (refer to Fig. 6).

### Rate of revisions

**Fig. 7:**
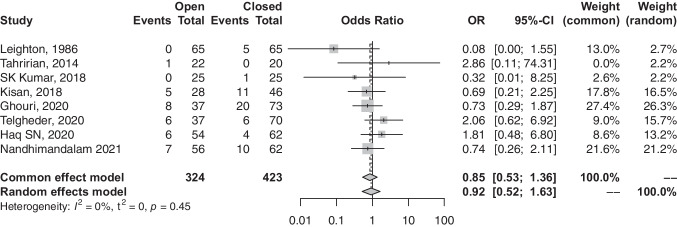
Forest plot comparison of revision rate between open and closed groups. OR, odds ratio; CI, confidence interval; Event, number of revisions; total, all treated fractures

In contrast, patients undergoing closed-reduction surgeries were associated with a higher probability with revisions (OR, 0.85; 95% CI, 0.53–1.35; *p*-value, 0.4987) (refer to Fig. 7). While the difference in revision rate was insignificant, heterogeneity was low among the 8 studies (*I*^2^, 0.0%; *p*-value, 0.4497).

### Publication bias

Egger’s test of bias demonstrated insignificant publication bias for dichotomous variables (*p*-value, 0.6913) (refer to Supplementary Funnel figures 1 through 5).

## Discussion

The main findings of this systematic review and meta-analysis were that union rate, nonunion rate, and infection rate were more favourable in the closed-reduction group, whereas the malalignment was more favourable in the open-reduction group. However, time to union and revision rates were comparable. A meta-analysis summary of the 6 main variable outcomes comparing open- versus closed-reduction methods is shown in Table 4.

**Table 4:** Meta-analysis summary of main outcomes comparing open- versus closed-reduction methods

Outcome	Open reduction	Closed reduction	OR or MD (95% CI)	Heterogeneity (I^2^)	*p*-value
	Effect	Effect			
Union rate (Fig. 2)	424	610	OR: 0.66; 95% CI, 0.45–0.97	34%	**0.0352**
Time to union (Fig. 3)	578	763	SMD: 0.41; 95% CI, − 0.11 to 1.21	90%	0.0906
Non-union (Fig. 4)	439	616	OR: 2.06; 95% CI, 1.23–3.44	0%	**0.0056**
Malalignment (Fig. 5)	442	560	OR: 0.32; 95% CI, 0.16–0.64	0%	**0.0012**
Infection rate (Fig. 6)	409	508	OR: 1.94; 95% CI, 1.16–3.25	0%	**0.0114**
Revision rate (Fig. 7)	324	423	OR: 0.85; 95% CI, 0.53–1.35	0%	0.4987

Bold emphasis statistically significant values (*p*-value < 0.05)

*OR*, odds ratio; *MD*, mean difference; *CI*, confidence interval; *Effect*, common-effect model

The intramedullary nail is considered the treatment of choice for nearly all femur shaft fractures. Ideally, fracture reduction is achieved by closed-reduction means; however, open reduction might be inevitable in certain situations where we have soft tissue interposition, severely comminuted fracture, and associated injuries, and in obese or muscular patients. Many studies have compared the outcome of closed versus open reduction in femur shaft fracture, with some authors claiming that the outcome is comparable between each group.

Contrary to several studies which showed no significant difference in union rate [16, 17, 19, 20], our analysis demonstrated that patients with femur shaft fractures treated with open reductions IMN had a significantly lower union rate when compared to those treated with closed reduction (OR, 0.66; 95% CI, 0.45–0.97). The violation and disruption of the fracture haematoma and its positive role in bone healing might explain this significant finding [26]. Similarly, a significantly higher non-union rate was observed in the open-reduction group (OR, 2.06; 95% CI, 1.23–3.44). Non-union was consistently defined across the included studies as the radiographic persistence of a radiolucent line without progression of callus formation, along with pain at the fracture site at a minimum of six months after the surgery. This universal agreement on defining non-union enabled us to pool these studies and compare this outcome with very low heterogeneity (*I*^2^ = 0%) and statistical significance. Telgheder et al. [21] retrospectively studied 107 patients with traumatic femur shaft fracture who underwent intramedullary nail preceded by either closed or open reduction; they reported a comparable mean time to union in closed reduction and open reduction and combined groups of 5.4 months, 6.2 months, and 5.6 months, respectively (*p* = NS). This present study supports this finding as there was no difference in time to union among both groups (SMD, 0.41; 95% CI, − 0.11 to 1.21, *p* = NS).

Karaman et al. [27] reported a 41.7% incidence of rotational malalignment of more than 10° following closed reduction and intramedullary nailing of femur shaft fracture on CT; these patients were symptomatic and had significantly lower functional scores compared to those without malalignment. Open-reduction techniques can aid in the precise and anatomical restoration of fracture fragments. Similarly, our meta-analysis showed that open reduction reduces the risk of malalignment to less than one-third compared to the closed-reduction group (OR, 0.32; 95% CI, 0.16–0.64, *p* < 0.05).

Infection rates following intramedullary nails of femoral shaft fractures are generally low, ranging from 1 to 3.8% [28]. Out of 118 enrolled patients, Nandhimandalam et al. [23] reported 4 cases of superficial infection, 2 in each arm and only one with deep infection in the open group, results which were statistically insignificant. These findings were also replicated by subsequent studies [20, 21]. Haq et al. [22] compared the infection rate in closed and open groups and reported a significantly higher risk with open reduction (6.4% vs 24%). However, pooling various studies demonstrated a twice higher risk of infection in patients with open reduction than those who underwent closed reduction (OR, 1.94; 95% CI, 1.16–3.25, *p* < 0.05). The increased surgical time, peri-operative antibiotics use, and direct manipulation of soft tissue through open reduction can contribute to this increased infection risk. However, these confounding factors were not clearly mentioned in the included studies. Thus, a future prospective study matching these confounders is warranted.

Although the revision rate difference was insignificant between the two groups, with a higher probability of revision in the closed-reduction group (OR, 0.85; 95% CI, 0.53–1.35), it is worth mentioning that the revision is done for all causes necessitating surgery, including but not limited to deep infection, nonunion, and malalignment [13, 21, 29].

To the best of our knowledge, this systematic review was the first to analyze and compare open- versus closed-reduction techniques in IMN treatment of femur shaft fractures. However, several limitations must be acknowledged. First, some of the included studies were of low quality, reducing the confidence in the results, including an RCT with a questionable randomization method and an apparent selection bias. Regardless, these studies remain the highest level of available evidence in the literature. Another weakness was the inadequate reporting of some confounding factors, such as the type of IMN used and baseline comorbidities which were not clearly stated in all studies. Also, several comparison outcomes, like operative time and radiation exposure, were either missed or poorly reported. Thus, future prospective studies are needed to adjust for these confounders.

## Conclusion

Closed-reduction IMN was associated with more favourable union rate, nonunion rate, and infection rate compared to the open-reduction group, whereas malalignment was significantly less in the open-reduction group. However, time to union and revision rates were comparable. This finding should be applied in context due to potential confounding factors and the lack of high-quality studies.

### Supplementary Information

Below is the link to the electronic supplementary material.

Supplementary file1 (DOCX 126 KB)

## Data Availability

Not applicable as this is a review article. However, happy to provide access to any statistical data (coding) upon request.
