# Supplementary material for: Open versus closed intramedullary nailing of femur shaft fractures in adults: a systematic review and meta-analysis
Source: Int Orthop. 2023 Mar 3;47(12):3031–41. doi: 10.1007/s00264-023-05740-x (PMC10673735; doi:10.1007/s00264-023-05740-x)
Supplement: Supplementary file 1 — Supplementary file1 (DOCX 126 KB) [file 264_2023_5740_MOESM1_ESM.docx]

Supplementary figures:

Figure 1

Figure 2

Figure 3

Figure 4

Figure 5

Figure SMD
